# Finished Genome Sequences of *Xanthomonas fragariae*, the Cause of Bacterial Angular Leaf Spot of Strawberry

**DOI:** 10.1128/genomeA.01271-16

**Published:** 2016-11-10

**Authors:** Peter M. Henry, Johan H. J. Leveau

**Affiliations:** Department of Plant Pathology, University of California Davis, Davis, California, USA

## Abstract

*Xanthomonas fragariae* is a foliar pathogen of strawberry that is of significant concern to nursery production of strawberry transplants and field production of strawberry fruit. Long-read sequencing was employed to generate finished genomes for two isolates (each with one chromosome and two plasmids) from symptomatic plants in northern California.

## GENOME ANNOUNCEMENT

*Xanthomonas fragariae* causes angular leaf spot (ALS) of strawberry (*Fragaria* × *ananassa*) and is an international quarantine pathogen (1, 2). This species of Gram-negative bacteria colonizes strawberry foliage and enters the leaves through wounds or stomata (3), where it may remain quiescent before initiating growth and the profuse production of exopolysaccharides which is associated with typical water-soaked leaf lesions (2, 3). ALS symptoms may be mitigated by the use of certified disease-free planting stock or the foliar application of copper (1). A draft genome of a Belgian *X. fragariae* strain (LMG 25863) was published previously (4). The abundance of insertion sequences on this genome greatly complicated the assembly of Illumina reads (draft status at 96 contigs). To obtain a complete reference genome for future resequencing projects, long-read sequencing technology (PacBio) was used on two strains of *X. fragariae* (*Fa*P21 and *Fa*P29) isolated in 2011 from symptomatic strawberry leaf tissue in Siskiyou County, California.

Genomic DNA was extracted from cells growing exponentially in liquid Wilbrinks-N (5) using a DNeasy blood and tissue kit (Qiagen, Valencia, CA). PacBio SMRTbell libraries were prepared at the UC Davis DNA Technologies Core (Davis, CA), size-selected to >20-kb fragments with BluePippin (Sage Science, Beverly, MA), and sequenced on the PacBio RS II platform (Pacific Biosciences, Menlo Park, CA). Reads were assembled by the hierarchical genome assembly process (HGAP3) protocol in smrtanalysis v2.3.0 (6) to yield for each isolate a single chromosome-length contig and two plasmid contigs. Ends of each contig were checked in Gepard (7) for overlapping regions that were trimmed and joined to yield complete circular chromosome and plasmid sequences (with the exception of one of the plasmids in *Fa*P29, which could not be circularized). The beginning of each circularized chromosome was set to the start codon of the *dnaA* gene. Assemblies were quality-checked with high-fidelity 150-bp, paired-end Illumina MiSeq reads (UC Davis DNA Technologies Core). Bowtie2 mapped 99.25% of reads to assemblies with mean coverages of >400× (8). Using Pilon (9), we corrected 33 and 39 indel errors in the *Fa*P21 and *Fa*P29 genomes, respectively. Gene prediction was done using the Rapid Annotation using Subsystem Technology (RAST) server (10).

The genomes of *X. fragariae Fa*P21 and *Fa*P29 were highly similar in size (4.2827 and 4.2826 Mbp, respectively), G+C content (62.27% for both), and number of RAST-predicted genes (4,149 and 4,141, respectively). The genomes harbored multiple gene clusters for copper resistance and an arsenal of type II, IV, VI, and VII secretion systems. Similar to LMG 25863 (4), genes for type III secretion and TAL effectors were absent in the *Fa*P genomes. The largest of the two plasmids (29.1 kb) in both strains showed homology to a 27.2-kb plasmid from the xylem-limited sugarcane pathogen *Xanthomonas albilineans*. Mapping of the 96 contigs of LMG 25863 onto the *Fa*P genomes revealed that the ends of these contigs consistently represented highly repeated regions on the genome, showing very clearly the benefit of using long-read technology to close bacterial genomes.

### Accession number(s).

The complete genome sequences for *Fa*P21 and *Fa*P29 have been deposited at DDBJ/EMBL/GenBank under the accession numbers CP016830 (*Fa*P21 chromosome), CP016831 (plasmid p*Fa*P21-1), CP016832 (plasmid p*Fa*P21-2), CP016833 (*Fa*P29 chromosome), CP016834 (plasmid p*Fa*P29-1), and CP016835 (plasmid p*Fa*P29-2).
